# Predictive Factors of Cerebral Aneurysm Rerupture After Clipping

**DOI:** 10.3389/fneur.2021.789216

**Published:** 2022-02-16

**Authors:** Yu-Jun Chang, Chi-Kuang Liu, Chun-Yuan Cheng, Yu-Cheng Shih, Shih-Chun Wang, Chung-Chih Lin, Chih-Ming Lin

**Affiliations:** ^1^Big Data Center, Epidemiology and Biostatistics Center, Changhua Christian Hospital, Changhua, Taiwan; ^2^Department of Medical Imaging, Changhua Christian Hospital, Changhua, Taiwan; ^3^Division of Neurosurgery, Department of Surgery, Changhua Christian Hospital, Changhua, Taiwan; ^4^Department of Post-Baccalaureate Medicine, College of Medicine, National Chung Hsing University, Taichung, Taiwan; ^5^Department of Neurology, Changhua Christian Hospital, Changhua, Taiwan; ^6^Graduate Institute of Statistics and Information Science, National Changhua University of Education, Changhua, Taiwan; ^7^Department of Research, Changhua Christian Hospital, Changhua, Taiwan

**Keywords:** cerebral aneurysm, aneurysmal clipping treatment, aneurysmal rerupture, aneurysm location, incidence

## Abstract

**Background:**

We aimed to estimate the risk of rerupture after first-time aneurysmal clipping surgery, explore the possible related factors, and assess long-term physical functionality. We hypothesized that the modified Rankin scale (mRS) could serve as an effective substitute for Hunter and Hess scale.

**Methods:**

This retrospective study included 171 patients with cerebral aneurysmal rupture who had completed aneurysmal clipping treatment and collected their demographic data and medical records. The outcome assessments include neuroimaging records, Hunter and Hess scale, and the mRS scale during hospitalization and follow-up after discharge. The mean length of follow-up was 4.28 years.

**Results:**

After aneurysmal clipping treatment, 83 patients (48.5%) had subsequently ruptured aneurysms. The scores of the reruptured group on the Hunt and Hess scale and mRS were significantly higher than those of the non-reruptured group. Multiple Cox proportional-hazards regression also showed that postoperative mRS >2, smoking, and two or more aneurysms were potentially important risk factors leading to aneurysm rupture again [the corresponding hazard ratios (HRs) were 5.209, 2.109, and 2.775, respectively] in patients. In addition, the location of an aneurysm on the anterior cerebral artery (ACA) or the posterior communicating (Pcom) artery had a higher risk of rerupture (the corresponding HRs were 1.996 and 2.934, respectively).

**Conclusions:**

Nearly half of the collected participants experienced the rerupture episode, who had undergone the second-time clipping surgery. Smoking and multiple aneurysms are potential risk factors for aneurysmal rerupture. Most aneurysms are located along the ICA, but aneurysms located at the ACA or Pcom site are most likely to rerupture. As compared with the Hunter and Hess scale, the mRS scale does not have inferior predicting power in following patients' long-term functionalities.

## Introduction

A cerebral aneurysm poses a life-threatening risk if not properly managed. Based on the statistics, cerebral aneurysm stands out as the third most disabling entity in the field of cerebral vascular diseases (1). The reason why it is so devastating is that the initial symptoms of cerebral aneurysm are not specific and often interpreted as the common flu, with a headache and dizziness as the most common initial presentations (2). As a result of this phenomenon, misidentification of the golden opportunity to diagnose and timely treatment at an early stage could be lost.

Upon reviewing the related literature (3, 4), it is clear that most articles focus on managing the outcomes of cerebral aneurysm in terms of cerebral location and size of the lesion before the operation and its subsequent outcomes. However, there is a lack of knowledge over the chance of rupture in patients undergoing aneurysmal clipping operation and the subsequent long-term functionality. Additionally, most aneurysms are found over the anterior circulation of the Circle of Willis, of which the reason why this is the case is yet to be studied in depth, although it has been postulated that it has something to do with blood flow fluctuations and plaque predilection over anterior circulation rather than in the posterior circulation (5). There has been little documentation of the mechanisms and weight of risk based on the various locations of aneurysms. A previous study found that compared with other locations, ICA aneurysms are lower in size ratios, have fewer hemodynamic characteristics, and also have a lower tendency to rupture. There was no significant statistical difference in the morphology or hemodynamic characteristics between the MCA aneurysms and the group of anterior communicating, posterior communicating, and posterior circulation aneurysms (6). Moreover, Hunt and Hess scale has traditionally been adopted as the gold standard outcome assessment tool. However, not all clinical medical personnel are familiar with this assessment tool, as it requires a certain level of neurological knowledge to carry out a correct evaluation.

In the current study, we aimed primarily to follow up the changes of patients with cerebral aneurysms and investigate the incidence of aneurysmal rupture after aneurysmal clipping treatment. Second, we sought the site of cerebral aneurysms with the greatest predilection and analyzed the possible causes of rupture. We are intrigued by risk factors among the baseline demographics, clinical, and imaging data. These factors can be early predictors of whether an aneurysm is ruptured or not after the aneurysmal clipping surgery, in terms of ensuring first-line clinicians receive red flag signals, that they can pay special attention to this group of patients. Finally, we also verified the diagnostic power of mRS to examine whether mRS could be served as an effective substitute for the Hunt and Hess scale in clinical practice.

## Materials and Methods

We retrospectively collected patients identified with cerebral aneurysmal rupture through neuroimaging studies from January 2012 to December 2016, with 171 total participants. The patients' list was completed and recorded at the Angiography Laboratory of the Department of Neuroimaging. The study protocol was approved by the research ethics committee of the hospital. Informed consent was waived as the patient personal information has been de-identified and is irreversible.

### Inclusion and Exclusion Criteria

We included age ≥20 years, ≤ 80 years, completion of at least 3 years of follow-up after angiography procedure (aneurysmal clipping treatment). We excluded patients with ischemic stroke, those with cerebral arteriovenous malformations during the study period, and those patients lost to follow-up. A total of 171 patients initially fulfilled the inclusion and exclusion criteria and were subsequently enrolled in the study. All patients underwent a comprehensive historical and physical examination, biochemistry, and neuroimaging investigations upon hospitalization, and their conditions were followed up after their discharge from the ward. Computed tomography and magnetic resonance imaging studies were arranged during admission. In addition, complementary examinations, including carotid duplex, digital subtraction angiography, and cardiac echography were also completed.

### Setting and Type of Study

This is a retrospective cohort study. We collected data from the medical records of consecutive patients who underwent cerebral angiography during the period January 2012 to December 2016 at the Angiography Laboratory of the Department of Neuroimaging of a medical center. The clinical information collected was cross-checked, including the paper form of medical charts and the electronic information of the hospital's computer-based system. We attempted to use the modified Rankin scale (mRS), a scale for the evaluation of patient outcome and long-term functionality, as an indicator to signal patient independence over the long term. The mRS was collected during admission and again during the outpatient setting. The evaluation time points were before the clipping operation, immediately after the operation, and 6 months after the operation.

The mRS is a commonly used scale for measuring the degree of disability or dependence in the daily activities of people who have suffered neurological disability from a stroke or other causes and has become the most widely used clinical outcome measure in clinical trials for stroke. The scale runs from 0 (perfect health without symptoms) to 6 (death). The information collected may improve our ability to prognosticate the outcome after cerebral aneurysmal clipping, to assist patients and their families with their decision regarding clipping surgery, and could also improve care and decision making in future studies of patients with cerebral aneurysmal.

### Cranial Computed Tomography

Multiple sequential axial images were obtained from the skull base up through the vertex without intravenous contrast material. The technical parameters were thickness, 5 mm; length, 200 mm; increment, 10 mm; kV, 120; mA, 550.

### Magnetic Resonance Imaging and Angiography

Structural and functional MR imaging and angiographic examinations were performed using a 3-T (Magnetom Verio, Siemens Healthcare, United States) or a 1.5-T imager (Magnetom Aera, Siemens Healthcare) with a cervical coil. The standard protocol for evaluating a stroke including axial DWI, apparent diffusion coefficient, and fluid-attenuated inversion–recovery sequences was followed. Three-dimensional TOF MR angiography without contrast enhancement was performed in the transverse plane using a sliding interleaved kY acquisition sequence comprising 6 overlapping slabs of 11 sections, with the following parameters: section thickness, 1.2 mm; repetition time (milliseconds)/echo time (milliseconds), 242/7; flip angle, 20°; field of view, 200 mm × 200 mm; matrix, 205 × 320. The final pixel size was 0. 975 mm × 0.625 mm. The entire imaging time was approximately 7 min. Contrast-enhanced MR angiography was not routinely performed.

### Digital Subtraction Angiography and Aneurysmal Clipping

The clipping protocol of our hospital is for all cerebral aneurysmal patients to have a digital subtraction angiography (DSA) examination before surgical clipping treatment to locate the cerebral aneurysm with precision. The same protocol also applies for the second operation or more rerupture participants in this study. Biplanar intra-arterial DSA was performed using a biplanar flap panel rotational angiography unit (Axiom Artis Zee, Siemens Healthcare) with an image intensifier matrix of 1,024 × 1,024 pixels and a final pixel size of 0.37 mm. Immediately after approaching the femoral artery, a 7-F catheter (Boston Scientific, Mach 1) was inserted into the right or left CCAs near the bifurcation. Posteroanterior and lateral projections were acquired at the level of the carotid bifurcation. A third oblique-angle projection was acquired if overlapping vessels were noted in the first 2 projections. For each projection, 11 mL of nonionic iodinated contrast medium (Omnipaque 350; GE Healthcare, Ireland) was intraarterially injected at a flow rate of 7 mL/s using an automatic injector (Mark V ProVis; Medrad). The location of the aneurysm was discussed by the neuro-radiologist with the family members. Subsequently, the patient was sent to the operating room for the clipping surgery to be carried out under general anesthesia conditions.

### Outcomes

The primary aim of this study was to evaluate the results of the follow-up of patients undergoing clipping treatment after cerebral aneurysm rupture and to calculate the rate of recurrence of aneurysm rupture after an operation, as well as to explore the possible causes of rerupture. We followed up with each patient from the day of the clipping operation to the day when the aneurysm ruptured again. In some cases, there was no rerupture up to and including the final day of the study of out-patient clinic follow-ups. The secondary outcome is to calculate the rate of rerupture of aneurysms in each artery and to identify the most common location of cerebral aneurysm rerupture. The mRS and Hunt and Hess scale were used to measuring the neurological function of patients, with the higher the scores of the two scales, the poorer the neurological function demonstrated.

### Statistical Analysis

Pre- and post-operation mRS scores were compared to determine patient outcomes. We divided the patients into a rerupture group and a non-rerupture group to compare their basic demographic data, pre-stenting exam variables, location of the aneurysm rupture, and changes in mRS. A Mann–Whitney U test was used to compare the median difference between the two groups of continuous variables, while a Pearson's Chi-square test or a Fisher's exact test was used to examining the difference in the frequency distribution of the categorical variables between the two groups. A receiver operating characteristic (ROC) curve was performed to measure diagnostic accuracy and identify which scale has better diagnostic power. Cumulative incidences of the cerebral aneurysmal rerupture after aneurysmal clipping treatment were estimated using the Kaplan–Meier method and the logrank test. In addition, univariate and multivariable Cox proportional hazards models were constructed to identify the potential risk factors for aneurysm rerupture and calculate the hazard ratios (HRs). All statistical analyses were performed using the statistical package SPSS for Windows (Version 22.0, IBM Corp, Armonk, NY, United States). *P*-values below 0.05 were considered to indicate statistical significance.

## Results

Of the 171 patients with aneurysmal rupture, 115 (67.3%) were women. The median age of the subjects was 61 years (range 27–88). Headache (52.6%) was the most common first symptoms presentation, followed by dizziness (49.1%), double vision (2.9%), and symptomatic epilepsy (2.3%). After aneurysmal clipping treatment and 5 years follow-up, 5 patients (2.9%) had a thromboembolic event, 83 patients (48.5%) had subsequently ruptured aneurysms, 13 patients (7.6%) had surgical retreatment, and 16 patients (9.4%) deaths. The median time from surgery to rupture of the aneurysm was 6.5 months (interquartile range 3.4–15.4). When comparing the comorbidity data for patients with reruptured and non-reruptured aneurysms, no significant differences were found, although the proportion of type 2 diabetes mellitus (DM) in the reruptured group was significantly lower than that in the non-reruptured group (9.6 vs. 26.1%, *P* = 0.005). As for either family history or operation history, our study shows no direct linkage. However, there was a higher percentage of people who smoked (16.9 vs. 8.0%) or had multiple aneurysms (12.0 vs. 4.5%) in the reruptured group than in the non-reruptured group (Table 1).

**Table 1:** Basic demographics of rerupture vs. Non-reruptured patients post aneurysmal treatment.

				**Aneurysmal rerupture**				***P*-value**
		**Total (*****n*** **=** **171)**		**No (*****n*** **=** **88)**		**Yes (*****n*** **=** **83)**		
		***N***	**%**	***N***	**%**	***N***	**%**	
Gender	Female	115	67.3	64	72.7	51	61.4	0.116
	Male	56	32.7	24	27.3	32	38.6	
Smoking	No	150	87.7	81	92.0	69	83.1	0.076
	Yes	21	12.3	7	8.0	14	16.9	
Alcohol	No	162	94.7	83	94.3	79	95.2	1.000
	Yes	9	5.3	5	5.7	4	4.8	
Comorbidity	Hypertension	87	50.9	48	54.5	39	47.0	0.323
	DM	31	18.1	23	26.1	8	9.6	0.005
	Hyperlipidemia	15	8.8	11	12.5	4	4.8	0.076
	Ischemic stroke	7	4.1	4	4.5	3	3.6	1.000
	Hemorrhage stroke	26	15.2	13	14.8	13	15.7	0.871
	Old coronary artery disease	13	7.6	8	9.1	5	6.0	0.449
	CHF	4	2.3	2	2.3	2	2.4	1.000
	Arteriovenous malformation	1	0.6	1	1.1	0	0.0	1.000
	Polycystic kidney disease	2	1.2	2	2.3	0	0.0	0.497
	CKD	3	1.8	3	3.4	0	0.0	0.246
	Hereditary disease	6	3.5	3	3.4	3	3.6	1.000
Family history	Aneurysm	24	14.0	11	12.5	13	15.7	0.552
	DM	12	7.0	5	5.7	7	8.4	0.481
	Hypertension	13	7.6	7	8.0	6	7.2	0.858
Operation history	No	122	71.8	62	71.3	60	72.3	0.882
	Yes	48	28.2	25	28.7	23	27.7	
Symptom	Dizziness	84	49.1	42	47.7	42	50.6	0.707
	Headache	90	52.6	44	50.0	46	55.4	0.478
	Diplopia	5	2.9	4	4.5	1	1.2	0.369
	Seizure	4	2.3	0	0.0	4	4.8	0.053
Multiple aneurysms	1	157	91.8	84	95.5	73	88.0	0.074
	2	14	8.2	4	4.5	10	12.0	

*P-value by Chi-square test or Fisher's exact test when appropriated*.

We further compared the physical condition and size of the aneurysm. It was found that there was no significant statistical difference between the two groups in terms of age, BMI, square, or the longest diameter of the aneurysms. In contrast, the Hunt and Hess scale, preoperative (pre-OP) mRS, immediate postoperative (post-OP) mRS, and follow-up post-OP mRS, and ΔmRS (change in mRS score 6 months after aneurysmal clipping treatment) scores in the reruptured aneurysm group were higher than those in the non-reruptured group (*P* < 0.001) (Table 2). In particular, aneurysms with poor pre-OP mRS, post-OP mRS, and followed up post-OP mRS score (>2) seem to have a higher tendency to rerupture (*P* < 0.001) (Table 3).

**Table 2:** Basic demographics of rupture vs. Non-ruptured patients as correlated with various outcome assessment tools.

				**Aneurysmal rerupture**						***P*-value**
	**Total (*****n*** **=** **171)**			**No (*****n*** **=** **88)**			**Yes (*****n*** **=** **83)**			
	**Median**	**Q_**1**_**	**Q_**3**_**	**Median**	**Q_**1**_**	**Q_**3**_**	**Median**	**Q_**1**_**	**Q_**3**_**	
Age	61.0	51.0	70.0	61.0	51.0	70.0	61.0	50.0	72.0	0.875
BMI	24.3	22.0	26.6	23.9	22.0	25.8	24.9	22.2	27.8	0.060
Square (mm^2^)	19.5	9.2	43.7	17.6	7.6	44.6	22.5	11.9	42.9	0.383
Diameter (mm)	3.1	2.1	4.4	3.0	2.1	4.2	3.2	2.1	4.7	0.502
Hunt & Hess scale	2.0	1.0	3.0	2.0	1.0	3.0	2.0	2.0	4.0	<0.001
Pre-OP mRS	2.0	1.0	4.0	1.0	1.0	3.0	4.0	2.0	5.0	<0.001
Post-OP mRS (immediate)	3.0	1.0	4.0	2.0	1.0	3.0	4.0	3.0	5.0	<0.001
Post-OP mRS (6 months later)	1.0	0.0	4.0	1.0	0.0	2.5	3.0	1.0	5.0	<0.001
ΔmRS (6 months later)	−2.0	−4.0	−1.0	−1.0	−3.0	0.0	−3.0	−4.0	−2.0	<0.001

*Q_1_ = Percentile 25; Q_3_ = Percentile 75*.

*ΔmRS (6 months later) = Post-OP mRS (6 months later) - Pre-OP mRS*.

*P-value by Mann–Whitney U-test*.

**Table 3:** Analysis of various cerebral aneurysm locations of two collected groups.

		**Total**	**Aneurysmal rerupture**				***P*-value**
			**No**		**Yes**		
			***N***	**%**	***N***	**%**	
	Total	171	88	51.5	83	48.5	
Multiple aneurysms	1	157	84	53.5	73	46.5	0.074
	2	14	4	28.6	10	71.4	
Aneurysm location							
ICA	No	88	34	38.6	54	61.4	0.001
	Yes	83	54	65.1	29	34.9	
ACA	No	144	81	56.3	63	43.8	0.004
	Yes	27	7	25.9	20	74.1	
MCA	No	151	80	53.0	71	47.0	0.275
	Yes	20	8	40.0	12	60.0	
PCA	No	162	83	51.2	79	48.8	1.000
	Yes	9	5	55.6	4	44.4	
Basilar	No	169	87	51.5	82	48.5	1.000
	Yes	2	1	50.0	1	50.0	
VA	No	157	81	51.6	76	48.4	1.000
	Yes	14	7	50.0	7	50.0	
AICA/PICA	No	165	83	50.3	82	49.7	0.212
	Yes	6	5	83.3	1	16.7	
Acom	No	167	88	52.7	79	47.3	0.053
	Yes	4	0	0.0	4	100.0	
Pcom	No	163	87	53.4	76	46.6	0.031
	Yes	8	1	12.5	7	87.5	
Pre-OP mRS	< =2	94	62	66.0	32	34.0	<0.001
	>2	77	26	33.8	51	66.2	
Post-OP mRS (immediate)	< =2	72	56	77.8	16	22.2	<0.001
	>2	99	32	32.3	67	67.7	
Post-OP mRS (6 months later)	< =2	101	66	65.3	35	34.7	<0.001
	>2	70	22	31.4	48	68.6	
Post-OP mRS (6 months later)	No Improved	27	24	88.9	3	11.1	<0.001
	Improved	144	64	44.4	80	55.6	
Thromboembolic event	No	166	84	50.6	82	49.4	0.369
	Yes	5	4	80.0	1	20.0	
Mortality	No	155	83	53.5	72	46.5	0.089
	Yes	16	5	31.3	11	68.8	
Retreatment	No	158	81	51.3	77	48.7	0.858
	Yes	13	7	53.8	6	46.2	

*P-value by Chi-square test or Fisher's exact test when appropriate*.

**Figure 1:**
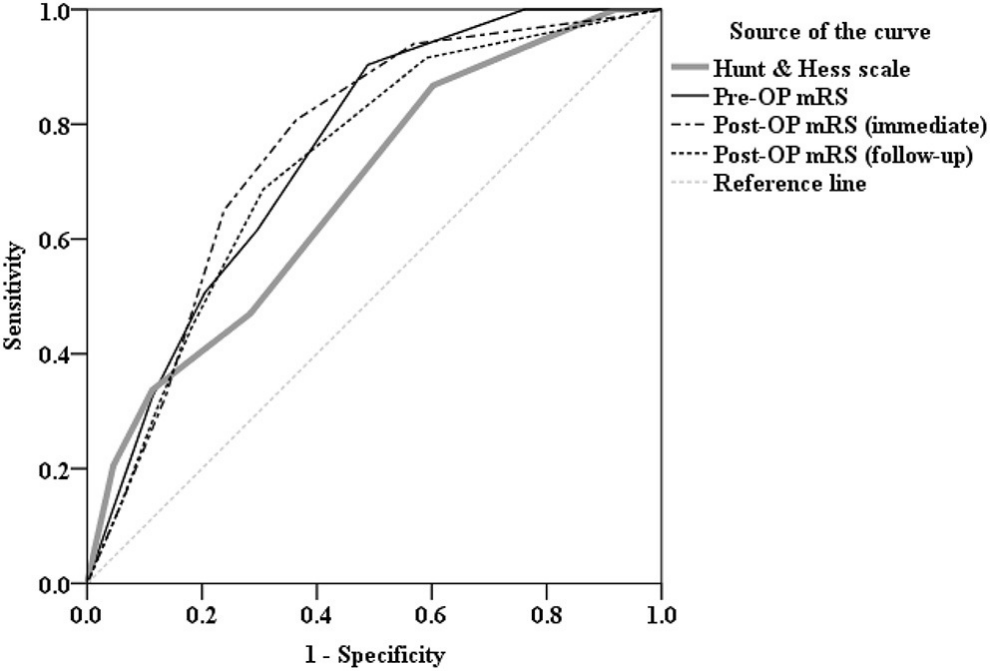
Receiver operating characteristic (ROC) curve analysis comparing the diagnostic power of Hunt and Hess scale [area under the curve (AUC):0.681, *P* < 0.001], Pre-OP mRS (AUC: 0.752, *P* < 0.001), Post-OP mRS (immediate) (AUC:0.760, *P* < 0.001), and Post-OP mRS (followed up) (AUC:0.731, *P* < 0.001) parameters in predicting whether there is subsequent rupture of an aneurysm after aneurysmal clipping treatment. As shown in the ROC curve, mRS, in particular, Post-OP mRS (immediate) stands out as the most powerful predictor and non-inferior to Hunt and Hess scale.

We then performed ROC curve analysis to compare the diagnostic power of Hunt and Hess scale, pre-OP mRS, post-OP mRS (immediate), and post-OP mRS (followed up) parameters in predicting whether subsequent rupture of an aneurysm after aneurysmal clipping treatment. As shown in the ROC curve, it revealed Hunt and Hess scale as area under the ROC curve (AUC): 0.681 (95% CI = 0.602–0.760, *P* < 0.001), pre-OP mRS as AUC: 0.752 (95% CI = 0.680–0.824, *P* < 0.001), post-OP mRS (immediate) as AUC: 0.760 (95% CI = 0.687–0.833, *P* < 0.001), and post-OP mRS (followed up) as AUC: 0.731 (95% CI = 0.655–0.806, *P* < 0.001); post-OP mRS (immediate) stands out particularly as the most powerful predictor and non-inferior to Hunt and Hess scale (Figure 1).

**Figure 2:**
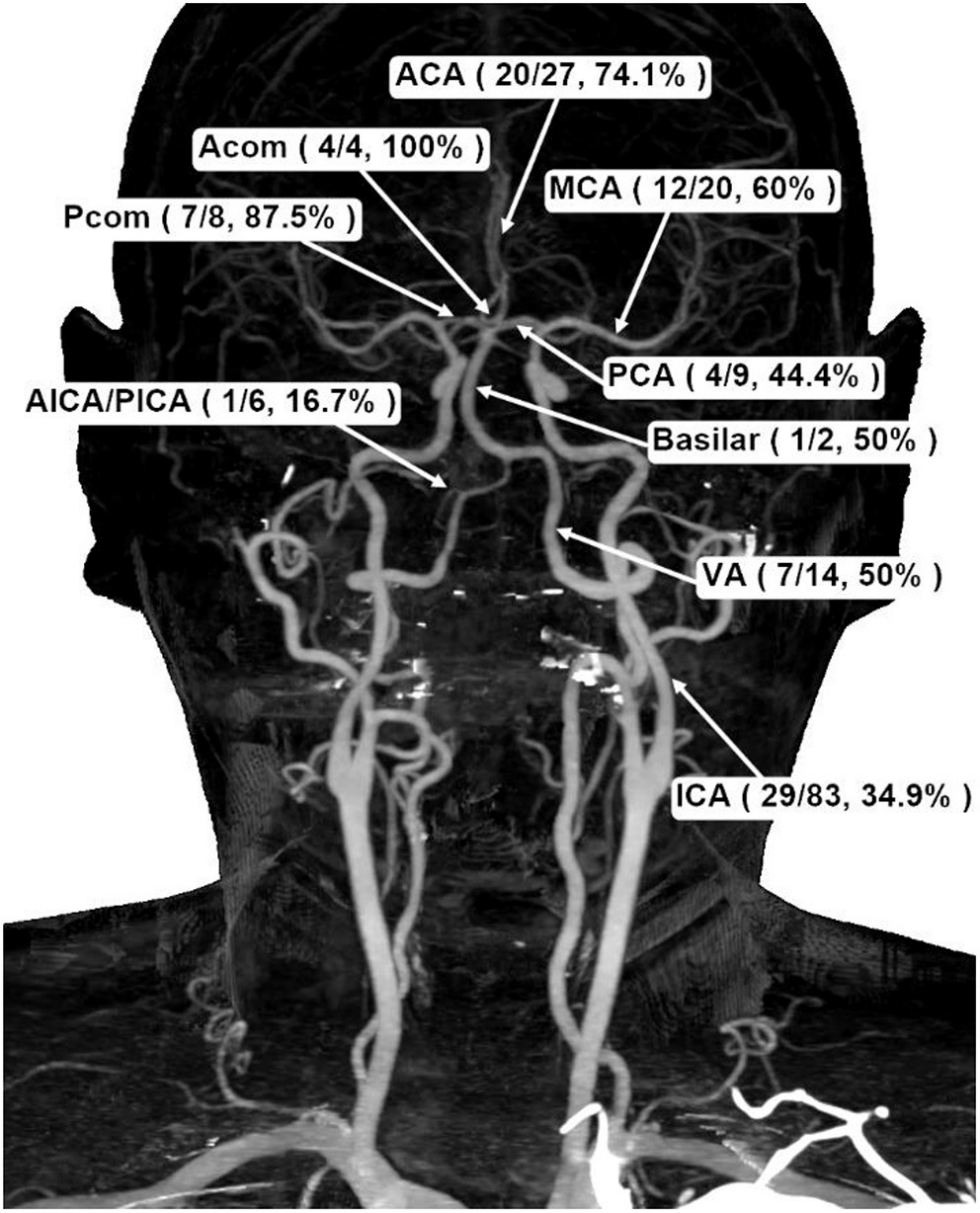
Schematic demonstration of aneurysmal frequencies and rerupture percentages in different cerebral basal artery locations. ACA, anterior cerebral artery; PCA, posterior cerebral artery; MCA, middle cerebral artery; Acom, anterior communicating artery; Pcom, posterior communicating artery; ICA, internal carotid artery; VA, vertebral artery; AICA/PICA, anterior inferior cerebellar artery/posterior inferior cerebellar artery.

Table 3 shows the location of aneurysms and the percentage of reruptures. In terms of intracranial aneurysm, terminal internal carotid artery (ICA) is the most common site (48.5%) where aneurysms are found during neuroradiological examinations, followed by anterior cerebral artery (ACA) (15.8%), middle cerebral artery (MCA) (11.7%), and posterior cerebral artery (PCA) (5.3%). However, of the anterior circulation, compared with ICA (34.9%), both MCA (60.0%) and ACA (74.1%) had more reruptures. As for the posterior circulation, there were fewer reruptures of aneurysms in PCA (44.4%) as well (Figure 2).

**Figure 3:**
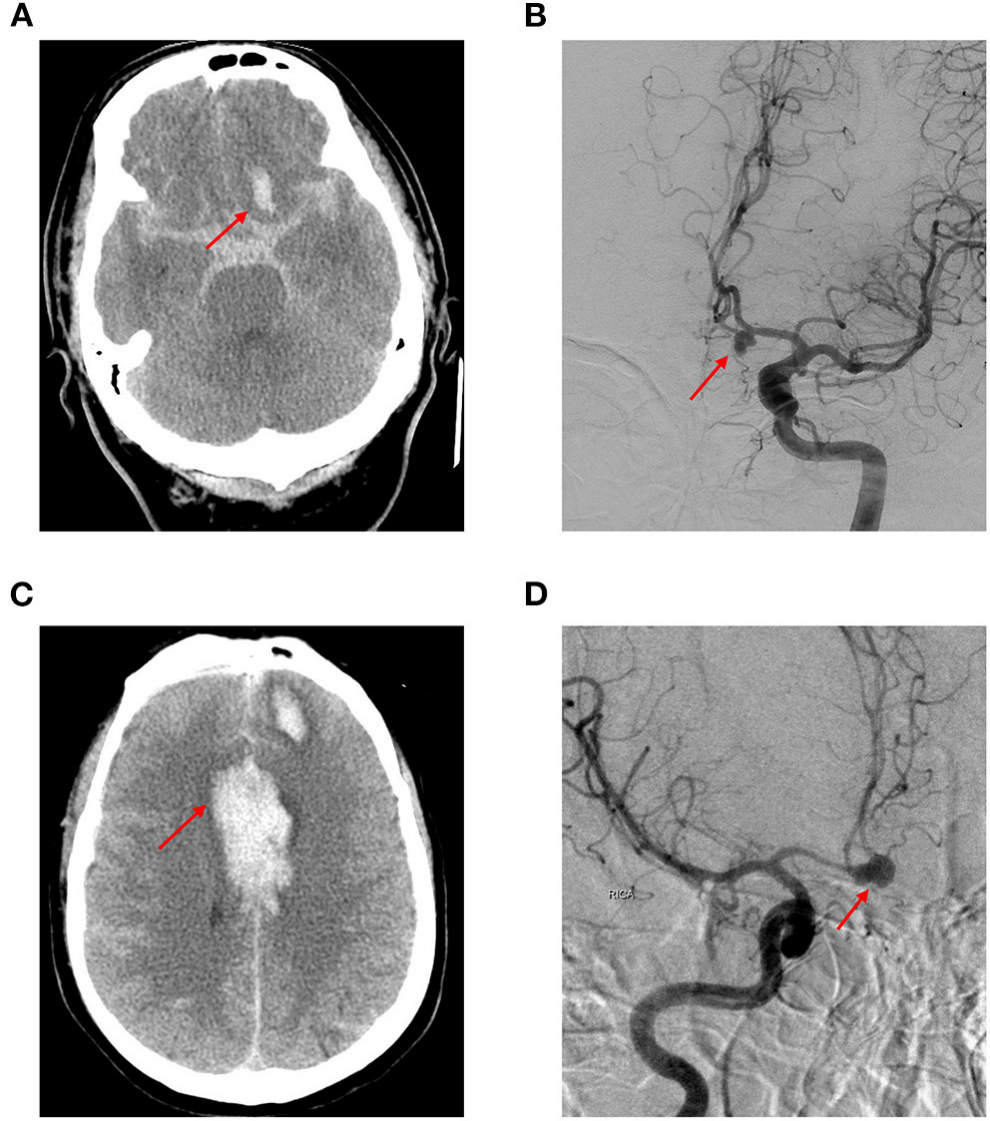
A 62-year-old Taiwanese woman presented to our emergency department with an abrupt and thunderclap headache. Initial cerebral computed tomography showed diffuse subarachnoid hemorrhage. **(A)** The subsequent digital subtraction angiography confirmed anterior communicating artery aneurysm **(B)**; she was sent for clipping surgery and discharged 1 month after the condition was stabilized. Eight months later, she was readmitted due to altered consciousness during the routine morning jogging. Cerebral computed tomography showed intra-cerebral hemorrhage and subarachnoid hemorrhage **(C)**; subsequent digital subtraction angiography demonstrated aneurysmal ruptured at the same location **(D)** (red arrow).

a) Upon receiving their first medical attention, all 83 patients presented with a cerebral rupture of SAH (Figure 3).

b) A total of 81 patients exhibited the same location rerupture status upon admittance to the hospital for DSA and subsequent clipping surgery, while 2 patients demonstrated a different location (one was in the anterior cerebral artery and the other was in the Pcom artery).

c) All of the patients, on both first and second admission to the hospital, underwent a DSA angiography diagnostic exam before surgery, as per hospital treatment protocol.

d) There were 10 patients with multiple aneurysms (2 or 3 locations) exceeding 8 mm in diameter.

e) It is our hospital protocol to perform DSA before intracranial neurosurgical clipping treatment, and no additional patient angiography was arranged or performed between two examinations. Based on the results of both DSA, we observed no aneurysms in new locations but an increase in the sizes of the original aneurysms before the operation the second time *via* digital subtraction angiography.

**Figure 4:**
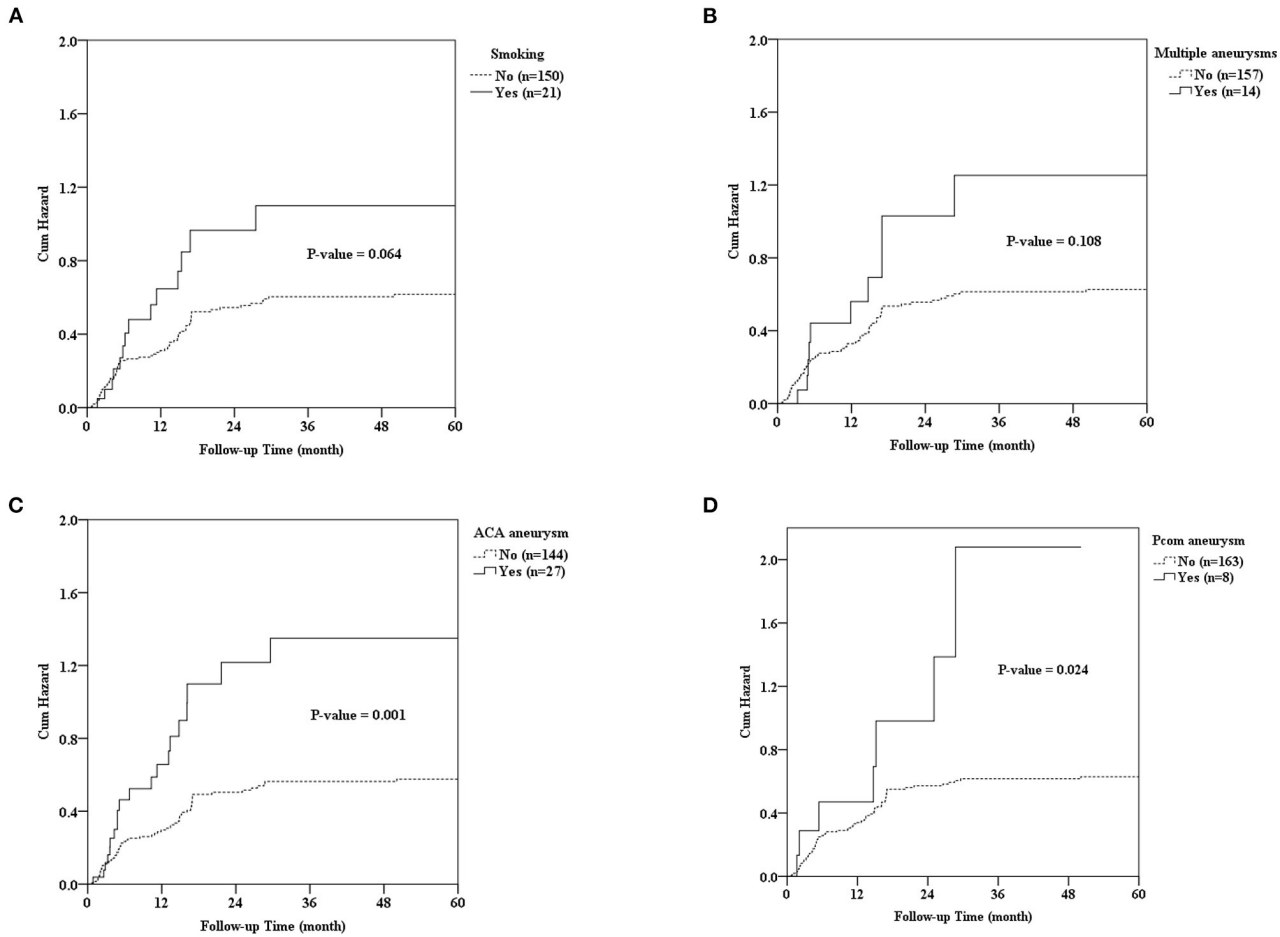
Cumulative incidence curves of aneurysmal rerupture after aneurysmal clipping treatment were estimated using the Kaplan–Meier method and the log rank test, stratified by **(A)** smoking habit, **(B)** multiple aneurysms, **(C)** ACA aneurysm, and **(D)** Pcom aneurysm.

We applied the Kaplan–Meier method and the logrank test to estimate the cumulative incidence of aneurysmal rerupture after aneurysmal clipping treatment. During the 5 years follow-up, the overall incidence density of aneurysmal rerupture was 12.6/1,000 person-months. We found that patients with aneurysms in the posterior communicating (Pcom) artery had the highest incidence of aneurysm rerupture (49.0/1,000 person-months), followed by patients with aneurysms in ACA (36.8/1,000 person-months). In addition, patients with smoking habit, multiple aneurysms, or post-OP mRS scores >2 also had a higher incidence of aneurysm rerupture (24.0, 30.2, and 23.8 per 1,000 person-months, respectively) (Table 4 and Figure 4).

**Table 4:** Cox proportional–hazards regression analysis of significant predictor in cerebral aneurysmal rerupture.

		***N***	**Person months**	**Rerupture**		**Bivariable analysis (crude)**			**Multivariable analysis (adjusted)**		
				**Event**	**Incidence**	**HR**	**95% C.I**.	***P*-value**	**HR**	**95% C.I**.	***P*-value**
DM	No	140	4897	75	15.3	1.000					
	Yes	31	1667	8	4.8	0.398	0.192–0.825	0.013			
Smoking	No	150	5981	69	11.5	1.000			1.000		
	Yes	21	583	14	24.0	1.713	0.963–3.047	0.067	2.109	1.170–3.799	0.013
Multiple aneurysms	1	157	6232	73	11.7	1.000			1.000		
	2	14	332	10	30.2	1.709	0.881–3.314	0.113	2.775	1.378–5.592	0.004
Pre-OP Hunt and Hess scale	≤ 2	107	4435	44	9.9	1.000					
	>2	64	2129	39	18.3	1.652	1.073–2.544	0.023			
Pre-OP mRS	≤ 2	94	4284	32	7.5	1.000					
	>2	77	2279	51	22.4	2.450	1.572–3.819	<0.001			
Post-OP mRS (immediate)	≤ 2	72	3748	16	4.3	1.000			1.000		
	>2	99	2816	67	23.8	4.303	2.488–7.442	<0.001	5.209	2.922–9.288	<0.001
Post-OP mRS (6 months later)	≤ 2	101	4572	35	7.7	1.000					
	>2	70	1991	48	24.1	2.586	1.670–4.006	<0.001			
ICA	No	88	2703	54	20.0	1.000					
	Yes	83	3860	29	7.5	0.491	0.313–0.773	0.002			
ACA	No	144	6021	63	10.5	1.000			1.000		
	Yes	27	543	20	36.8	2.261	1.363–3.751	0.002	1.996	1.190–3.349	0.009
Pcom	No	163	6421	76	11.8	1.000			1.000		
	Yes	8	143	7	49.0	2.372	1.092–5.151	0.029	2.934	1.313–6.556	0.009

*HR, hazard ratio; Incidence, per 1,000 person-months*.

We further conducted Cox proportional–hazards regression analyses to identify the risk factors of aneurysm rerupture. The results of univariate regression analysis still showed a significant negative correlation between type 2 diabetes mellitus (DM) and aneurysm rerupture (HR = 0.398, *P* = 0.013). People with DM seem to have a protective effect compared to those without DM, and their aneurysms are less likely to rupture. With smoking patients, the risks for aneurysms to rerupture were found to be 1.713 times higher and for multiple aneurysms 1.709 times higher the risk. Furthermore, for every 1 point increase in the mRS score (pre-op or post-Op) and Hunt and Hess scale, the risk of aneurysm rerupture increased by more than 40%. Aneurysms located on the anterior cerebral artery (ACA) or Pcom artery also had a higher risk of rerupture (the corresponding HR were 2.261 and 2.372, respectively), while aneurysms located on the ICA artery had a lower risk of rerupture (the HR was 0.491). After controlling for possible confounding factors, the results of multiple regression analysis showed that smoking, multiple aneurysms, post-Op mRS score (immediate), and the location of the aneurysm were independent predictors of aneurysm rerupture (Table 4).

## Discussion

This study aimed to provide important hints aiding clinical decision making in terms of early prediction of long-term functionality after aneurysmal clipping surgery. Most clinical decision making relating to ruptured aneurysms discusses the issue of the site of the aneurysm and the suitable cutoff point values to send the patients to clipping operation (7, 8). A few documented literature (5, 6) have stressed comparing the basic nature or the weighing of risks in terms of various locations of ruptured vs. non-ruptured aneurysms post aneurysmal clipping surgery, and they have not touched on the role of the easy- to-use mRS functionality test, as a substitute to the Hunt and Hess scale for early prediction of long-term functional outcomes after patient receiving an operation. A great portion of published articles (1, 3, 4, and 9) have stressed the appropriateness of aneurysmal clipping surgery based on the cerebral location and the size of the lesion. Nevertheless, long-term functional recovery and the chance of rerupture vary and are often neglected based on clinical observation (1).

In the current study, we hypothesized that mRS is an effective substitute in comparison to the Hunt and Hess scale in predicting long-term physical functionality outcomes. This notion arises from the neurological and neurosurgical wards, where most of the admitted patients are stroke patients. mRS is a commercially available and easy-to-use clinical assessment tool. By contrast, properly carrying out and documenting the Hunt and Hess scale requires some degree of neurological training and anatomical knowledge. In this study, we found that the mRS score is non-inferior to the Hunt and Hess scale in predicting the subsequent rupture of an aneurysm after aneurysmal clipping treatment (Figure 1). mRS testing was carried out after admittance to the hospital but before the operation, during the first week after the patient's discharge from the ward, and then 3, 6, and 12 months later. It is clear that mRS carried out before and after the operation can both predict and early predict the two groups' outcomes, a finding that is more scientifically objective when compared to the Hunt and Hess scale, where the patient is only tested before the operation. In the neurological ward, mastering mRS took only a few hours with medical staff and nursing staff trained to do the test. Comparisons of the two groups showed little difference in both timing and accuracy (Figure 1).

From Tables 1, 4, we have noticed that diabetes mellitus seems to be a protective factor when compared with non-diabetic patients, the percentage of rerupture in diabetic patients is lower (HR = 0.398, 95% CI: 0.192-0.825, *P* = 0.013); we surmise that because patients with DM may have other risk factors as well as they are likely taking daily anti-DM drugs as well as anti-hypertensive, anti-lipidemic, or even anticoagulant drugs to inhibit blood vessel atherosclerosis change or blood vessel obstruction, which reduces the chance of aneurysmal rupture. Unfortunately, we did not collect enough drug and relevant biochemistry datasets that could have provided more information for first-line doctors.

In Table 3, we dissect the clinical information for anterior circulation (MCA, ACA, ICA, and anterior communicating artery) and posterior circulation (basilar artery, bilateral vertebral arteries, and Pcom artery) for comparison of the reruptured and non-reruptured groups. The most common locations of the cerebral aneurysms were ICA (48.5%), ACA (15.8%), and MCA (11.7%). We also found that if the patient had either MCA or ACA aneurysm, the tendency to rerupture is more likely compared to ICA. The possible reason for this finding might be due to the fact that the terminal internal carotid artery is rather tortuous in the cranium. The long-term uncontrolled risk factor (essential hypertension, diabetes mellitus, etc.) might cause the blood vessels to dilate unexpectedly with the predisposition of the atheroma deposited over the inner layer of the intima media layer of the intracranial vasculature. Based on the normal intracranial basal arteries, mean flow velocity of the color-coded transcranial duplex study, the highest is MCA, followed by ACA and terminal ICA (9, 10). It is not surprising that MCA might cause an already existing entity to rerupture.

In the multiple regression analysis, we found that aneurysms located on the Pcom artery also have a very high risk of rerupture (HR = 2.934, 95% CI: 1.313–6.556, *P* = 0.009) (Table 4). Compared with the middle cerebral artery or the posterior cerebral artery, common aneurysm sites are the anterior communicating (Acom) and Pcom arteries, as Acom and Pcom are of small vessel calibers and prone to unstable systemic blood pressure leading to the formation of the aneurysm and subsequent rupture event (4, 5). In this study, since the case numbers of posterior circulation vessel datasets are few, more studies are required to validate this finding (11–13).

Moreover, post-Op mRS score (immediate) > 2 and smoking existence stand out as the important predictors for a tendency to rerupture (the corresponding HR were 5.209 and 2.109, respectively), leading to a poor neurological outcome (Table 4). For patients with cerebral aneurysmal rupture, if the mRS score does not return to normal after arterial clipping, we suspect that the patient still has other unresolved comorbidities that may cause the aneurysm to rupture again. As with the common risk factor for stroke patients, smoking might play a crucial role in determining the long-term outcome for a patient with aneurysmal. Most of the patients with a smoking habit might also have hypertension or diabetic mellitus. Drug intake combined with a nicotine component might be secreted in urine or stool.

Multiple aneurysms are also an important predictive factor, as patients with this condition have a higher chance of rerupture (HR = 2.775, 95% CI: 1.378–5.592, *P* = 0.004) (Table 4). For patients with multiple lesions in the cranium, the limited space might be compressed, leading to compression of the existent cerebral vasculature, causing systematically high blood pressure. In long-term circumstances such as this, the chance of rupture is naturally higher and more devastating than it is in those patients without this condition (14, 15).

Previous studies have suggested that cerebral aneurysms might be due to the intracranial idiopathic inflammation process. The success of clipping of the evident aneurysm shown on the neuroimaging study could not eliminate the underlying promoter matrix metallo-proteinases (MMPs) (1, 2, 16) function that might produce another cerebral lesion. In addition, based on the current consensus, the cerebral aneurysm ratio defined as height divided by neck diameter > 1.6 is considered the most powerful inclusion criteria for candidates of aneurysmal clipping surgery (17, 18). However, the post-surgery survival rate for patients in this group has not been meticulously investigated. In the current study, based on the basic demographics and long-term functional stratification analysis of patients, we compared reruptured and non-reruptured patients and calculated the incidence and risk of aneurysm rerupture after aneurysmal clipping treatment under various conditions. Some important clinical predictors found in this study could be provided for reference for first-line clinicians. We believe this study is the first to demonstrate the importance of predictors for those patients receiving aneurysmal clipping surgery to aggressively control the risk factors to, thus, avoid the need for a second operation.

All enrolled patients went through initial CT, MRI/MRA, and DSA (angiography) evaluation before surgical clipping treatment. For the rerupture cases, DSA was routinely arranged again to localize the rerupture site prior to the operation. In the current research, we observed an overall 48.5% of rerupture rate in our cohort study, in which this finding was in alignment with the guideline recommendation (19), that clipping surgery is more suitable for patients with tortuous basal artery structure.

Some limitations exist in the current study design, and these should be considered when interpreting the results. First, the current study was conducted in a single medical center facility; therefore, the replicability is limited to similar settings. The collected participants were of Asian origin, so external applicability should be cautiously made to other ethnicities. Second, the nature of the current study is retrospective in nature, therefore it is hard to fully monitor missing datasets or medication usage.

## Conclusions

Cerebral aneurysmal rupture is a potential medical entity that deserves special attention in terms of early diagnosis and prevention. In our study, nearly half of the aneurysmal patients experience the re-rupture episode and subsequent second-time clipping surgery. The current investigation also suggests that patients with a habit of smoking and with underlying multiple aneurysms should be treated more cautiously before their operation. Acom, Pcom, and ACA aneurysms pose a higher risk of rerupture tendency after aneurysmal clipping surgery, therefore, the patient should be closely monitored. Finally, mRS could be a useful clinical tool, in addition to the Hunt and Hess scale, in predicting patients' long-term functional outcomes after clipping surgery.

## Data Availability Statement

Data that support the findings of this study are available from the corresponding author, upon reasonable request.

## Ethics Statement

The studies involving human participants were reviewed and approved by the Research Ethics Committee, Changhua Christian Hospital. Written informed consent for participation was not required for this study in accordance with the national legislation and the institutional requirements.

## Author Contributions

Y-JC, C-KL, C-YC, Y-CS, and C-ML contributed to the design of the study. C-KL, C-YC, S-CW, and C-ML collected data. C-CL contributed to data management. C-YC, Y-CS, and S-CW validated the data. Y-JC and C-ML analyzed the data and drafted the manuscript and interpreted the data. All authors critically reviewed and edited the manuscript.

## Conflict of Interest

The authors declare that the research was conducted in the absence of any commercial or financial relationships that could be construed as a potential conflict of interest.

## Publisher's Note

All claims expressed in this article are solely those of the authors and do not necessarily represent those of their affiliated organizations, or those of the publisher, the editors and the reviewers. Any product that may be evaluated in this article, or claim that may be made by its manufacturer, is not guaranteed or endorsed by the publisher.
